# Isolation of Antagonistic Bacterial Strains and Their Antimicrobial Volatile Organic Compounds Against *Pseudogymnoascus destructans* in *Rhinolophus ferrumequinum* Wing Membranes

**DOI:** 10.1002/ece3.71628

**Published:** 2025-06-27

**Authors:** Yanqing Da, Mingxuan Liu, Yangshuang Zhu, Weixu Wang, Yaping Lu, Keping Sun

**Affiliations:** ^1^ Jilin Provincial Key Laboratory of Animal Resource Conservation and Utilization Northeast Normal University Changchun China; ^2^ Key Laboratory of Vegetation Ecology Ministry of Education Changchun China

**Keywords:** *Pseudogymnoascus destructans*, *Rhinolophus ferrumequinum*, skin bacteria, volatile organic compounds

## Abstract

White‐nose syndrome (WNS), caused by the fungus *Pseudogymnoascus destructans* (*Pd*), has led to significant mortality and species endangerment in North America. Bats in eastern China, however, carry low loads of *Pd* and do not exhibit disease, suggesting natural resistance. To explore potential defenses, we isolated and screened antagonistic bacteria from the wing membranes of *Rhinolophus ferrumequinum* for their ability to inhibit *Pd* growth. We employed the plate delineation isolation method to obtain 74 single strains, which were then screened for antagonistic effects through contact and non‐contact inhibition experiments. A total of 18 antagonistic strains were isolated. After sequencing and comparison with the NCBI database, we identified eight known species and three unidentified species of antagonistic bacteria: *Pseudomonas carnis*, *Buttiauxella ferragutiae* , *Paraburkholderia fungorum*, *Arthrobacter rhombi* , *Serratia liquefaciens* , *Paeniglutamicibacter gangotriensis*, *Brevibacterium aurantiacum* , *Acinetobacter lactucae*, *Pseudomonas* sp., *Brevibacterium* sp., and *Acinetobacter* sp. Seventeen isolated strains showed varying degrees of inhibition by contact, while five species, including *P. carnis*, *B. ferragutiae* , *S. liquefaciens* , *P. gangotriensis*, and *Brevibacterium* sp., also inhibited *Pd* via non‐contact mode. We utilized solid‐phase microextraction coupled with GC–MS to obtain approximately 20 volatile compounds, all of which exhibited inhibitory effects on the growth of *Pd*, including ketones, aldehydes, and sulfur ethers. Notably, 5 ppm of 1‐undecene, dimethyl trisulphide, and 2‐nonanone each independently inhibited *Pd* growth. These findings suggest that the antagonistic strains and their VOCs might help protect bats from WNS. Understanding the interactions between *Pd* and skin flora, along with their VOCs, may be crucial in mitigating bats' vulnerability to WNS and developing effective mitigation strategies in the future.

## Graphical abstract

In this study, 18 antagonistic bacteria inhibiting *Pd* growth were isolated from *R. ferrumequinum* skin. Of these, 17 exhibited contact inhibition, while 7 showed non‐contact inhibition, and they were identified as belonging to 12 taxa. Among those exhibiting non‐contact inhibition, *B. ferragutiae* , *S. liquefaciens* , and *P. gangotriensis* were most effective. For contact inhibition, *P. carnis*, *A. rhombi* , *S. liquefaciens* , and an unidentified *Pseudomonas* species were most effective. Approximately 20 VOCs were isolated, with 5 ppm of 1‐undecene, dimethyl trisulfide, and 2‐nonanone showing significantly inhibitory effects on *Pd*. This research highlights the skin microbiota as a crucial defense against fungal pathogens and may offer new approaches and microbial sources for controlling *Pd* spread.

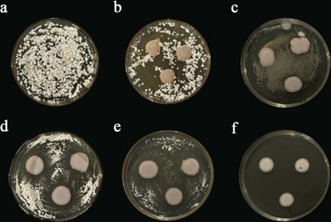

## 1 Introduction

Fungal pathogens have become a significant threat to vertebrate species and populations, often leading to the endangerment or extinction of affected species (Alangaden 2011; Rachowicz et al. 2005; Skerratt et al. 2007). One such pathogen, *Pseudogymnoascus destructans* (*Pd*), causes white‐nose syndrome (WNS), a fungal disease that has severely impacted hibernating bat populations since its discovery in 2006 (Mallinger et al. 2023). Bats infected with WNS typically show white mycelium of *Pd* on their mouths, ears, or wing membranes (Blehert et al. 2009). The colonization of *Pd* on the bat's wing membrane causes inflammation, which leads to premature awakening from hibernation, resulting in bats depleting their fat reserves and ultimately dying from starvation (Meteyer et al. 2009). The economic implications of WNS are significant, as insectivorous bats play a crucial role in pest control within agroforestry, and the resulting bat mortality is estimated to cost over $3.7 billion annually (Boyles et al. 2011). So far, *Pd* has been reported across several regions, including North America, Europe, and East Asia (Alves et al. 2014; Gugnani and Denning 2023; Wibbelt et al. 2010).

The prevention and control of white‐nose syndrome have garnered considerable attention from researchers. One suggested approach involves the use of antifungal drugs like gefitinib and anti‐EGFR antibodies to inhibit the growth and spread of *Pd* (Court et al. 2017; Isidoro‐Ayza and Klein 2024). Other studies propose creating warmer hibernation refuges for bats to reduce the frequency of awakening due to infection (Boyles et al. 2011). In addition to these strategies, the skin of animals, which serves as the first line of defense against pathogenic microbial invasion, has emerged as a key focus (Hou et al. 2022). Studies have highlighted the crucial role of symbiotic skin microbiota in protecting against fungal pathogens (Antwis and Weldon 2017; Conlon 2011; Lebaron and Bourrain 2017). For instance, Wan et al. (2024) isolated several genera from the skin of *Paramesotriton hongkongensis* , such as *Acinetobacter* and *Flavobacterium*, which displayed inhibitory effects against the fungus *Batrachochytrium dendrobatidis* (*Bd*). Similarly, Ange‐Stark et al. (2023) identified *Pd*‐antagonistic genera like *Fusobacterium* sp. in the wing membranes of North American bats. Li et al. (2022) also found that three strains of *Pseudomonas* isolated from bat wing membranes in China displayed strong resistance to *Pd*.

Actually, the metabolic activities of skin microbiota produce substances that demonstrate significant antagonistic effects against fungal pathogens (Ujszegi et al. 2023). For example, Woodhams et al. (2018) demonstrated that secondary metabolites, such as 2,4‐diacetylresorcinol, violetin, and indole‐3‐carboxaldehyde, produced by *Pseudomonas aeruginosa* and *Haemolyticus* sp., have a notable inhibitory impact on *Bd*. Similarly, Micalizzi et al. (2017) discovered that volatile organic compounds (VOCs), such as isobutanol, propionic acid, and 2‐methyl‐1‐butanol, effectively inhibited *Pd* growth. These findings suggest that the skin microbiota of bats could be a valuable resource for developing novel methods to combat white‐nose syndrome and protect bat populations from the devastating effects of *Pd*.

This study focuses on the bat species *Rhinolophus ferrumequinum* (Rhinolophidae, *Rhinolophus*), which is widely distributed across Asia and Europe. The species is classified as “Low Risk/Near Threatened (LR/NT)” by the IUCN, and its populations are facing endangerment in several European regions (Rossiter et al. 2000). In eastern China, *R. ferrumequinum* exhibits strict hibernation behavior, and its roosting sites maintain a temperature of around 10°C–13°C year‐round, which is similar to the optimal growth temperature for *Pd*. While the fungal load detected from this species was minimal, and infected individuals did not exhibit clinical symptoms of the disease, this suggests a high level of resistance to *Pd* (Hoyt et al. 2016). Additionally, two strains of *Pseudomonas* isolated from the body surface of hibernating *R. ferrumequinum* displayed the ability to inhibit *Pd* growth (Li, et al. 2022). However, due to the rich diversity of skin microbiota in *R. ferrumequinum* (Li, et al. 2022), studies screening for antagonistic strains remain limited. Thus, this study aims to screen for additional antagonistic strains found in the wing membranes of hibernating *R. ferrumequinum* in northeastern China. It will also detect the VOCs produced by these strains and assess their potential in resisting *Pd*. The study is expected to provide additional microbial sources and important VOCs for controlling WNS and help develop conservation strategies.

## 2 Materials and Methods

### 2.1 Sample Collection

In March 2021, nine *R* *. ferrumequinum* were collected from Fuyun Cave, Fangshan District, Beijing. The sampling site is located at the northern edge of the Taihang Mountains (115°59′N, 39°43′E), at an altitude of 900–1200 m, the cave's depth is about 1000 m, and the surrounding vegetation is abundant (Figure S1). Since *R. ferrumequinum* hibernate by wrapping themselves in their wing membranes, the dorsal skin might initially act as an inhibitor against *Pd*. The same side of each bat's dorsal wing membrane was wiped back and forth using a sterile cotton swab dipped in sterile water. The swabs were preserved in 30% glycerol for isolating and screening antagonistic strains. Samples for each bat were collected independently to prevent cross‐contamination. All samples were kept in an icebox until returned to the laboratory and then stored at −80°C.

### 2.2 Acquisition and Preservation of Single Colonies and Acquisition of *Pd* Spore Suspensions

Luria‐Bertani (LB) medium was used to culture and isolate bacteria from the wing membrane of *R. ferrumequinum* . Field‐collected samples were preserved in glycerol and then diluted 10‐fold and 100‐fold with sterile water to prepare bacterial suspensions. These suspensions were evenly spread on LB agar using an inoculation stick, with 50 μL applied per plate. Each concentration was prepared in triplicate and incubated at 13°C and 90% relative humidity (RH) for approximately 14 days, mimicking bat roosting conditions (Blehert and Lorch 2021). The method we chose to obtain single colonies was the streak plate method, where bacteria were streaked sequentially on the surface of the agar medium. This process ultimately results in a few bacterial cells being inoculated at the end of the final streak, leading to the formation of well‐distributed, independent colonies. Single colonies with distinct morphological characteristics were selected and subjected to three rounds of streak isolation on LB agar to obtain pure colonies. These colonies were then inoculated into LB liquid medium, cultured in a shaker at 13°C and 200 r/min, after 7 days, mixed with glycerol in a 1:2 ratio, and stored at −80°C.

*Pseudogymnoascus destructans* JHCN111a, preserved by the Key Laboratory of Conservation and Utilization of Animal Resources of Northeast Normal University, Jilin Province, was used in this study. It was first isolated from the body surface of bats in northeastern China in 2016, with an rRNA sequence identical to that of the pathogenic *Pd* strains from North America and Europe. Histopathological examination of bat wings has shown that it causes characteristic lesions of WNS (Hoyt et al. 2016). To collect *Pd* spores, they were inoculated onto Sabouraud Dextrose Agar (SDA) and allowed to grow for 3 weeks. Then, 10 mL of 1× Phosphate Buffered Saline with Tween 20 (PBST20) buffer was added to the medium. The spores were gently scraped off in one direction with a sterile inoculation loop. The mixture was shaken gently for about 5 min, then pipetted onto a cotton pad and filtered to obtain the *Pd* spore suspension. We determined spore concentration in the suspension using a hemocytometer, yielding a count of 6.5 × 10^5^/mL. After the streak plate step, we co‐cultured all the isolates obtained with *Pd* for contact inhibition and non‐contact inhibition experiments without any special selection for antagonistic screening.

### 2.3 Screening of Antagonistic Strains

#### 2.3.1 Contact Inhibition Screening

Contact inhibition experiments were performed by culturing isolates on SDA, where *Pd* was present (Hoyt et al. 2015). For the experimental group, 100 μL of 6.5 × 10^5^/mL *Pd* spore suspension was spread on SDA plates (90 mm ×18 mm). Then, 15 μL of bacterial suspension from isolated colonies was pipetted at three evenly spaced points in an equilateral triangle at the center of the plate. Control plates contained only *Pd*. All plates were incubated at 13°C and 90% RH in an incubator (XS‐ZD1, China), and *Pd* growth was assessed after 14 days. All assays were performed in triplicate.

Inhibitions were classified into five types according to Magan and Lacey (1984). Reaction type A: Bacteria intermingle with *Pd* and inhibit one or both colonies. Reaction type B: An inhibition halo of < 2 mm, indicating limited inhibition. Reaction type C: An inhibition halo > 2 mm, but inhibition is confined to this area. Reaction type D: Contact inhibition, where the inhibitor continues to grow either unchanged or at a reduced rate through the inhibited colony. Reaction type E: Distance inhibition, where the inhibitor grows through a clear zone and the inhibited colony, potentially at a reduced rate. Each type was assigned a score from 1 to 5. The total score for each isolated strain was calculated, and the mean inhibition score was determined based on three replicates.

#### 2.3.2 Non‐Contact Inhibition Screening

Non‐contact inhibition experiments followed the method of Rouissi et al. (2013). 100 μL of 6.5 × 10^5^/mL *Pd* spore suspension was spread on SDA agar plates (90 mm ×18 mm). Then, 20 μL of bacterial suspensions from isolates were inoculated onto LB agar plates and placed on top of the *Pd*‐coated SDA medium. The two plates were sealed with cellophane and incubated at 13°C and 90% RH. Control plates had only *Pd* on SDA under the same conditions. We observed *Pd* growth after 14 days in both experimental and control groups. All assays were performed in triplicate.

### 2.4 Sequence Comparison of Antagonistic Strains

The bacterial 16S rRNA gene was used to identify the antagonistic strain species, which were obtained by inhibition screening. PCR amplification was performed using primers 27F (5′‐AGAGTTTGATCCTGGCTCAG‐3′) and 1492R (5′‐GGTTACCTTGTTACGACTT‐3′). The PCR reaction mixture comprised 25 μL, including 1 μL of DNA template, 12.5 μL of 2 × PCR Master Mix (Sangon Biotech Co. Ltd., Shanghai, China), 1 μL each of forward and reverse primers, and 9.5 μL of double‐distilled water (ddH_2_O). A single bacterial colony was suspended in 100 μL of sterile water, boiled for 10 min, and used as the DNA template. PCR conditions were as follows: initial denaturation at 94°C for 5 min, followed by 35 cycles of 94°C for 1 min, 51.4°C for 45 s, 72°C for 1 min, with a final extension at 72°C for 10 min.

PCR products were detected using gel electrophoresis on 1% agarose. The target fragments were purified and recovered using a DNA gel recovery kit (EZ‐10 Spin Column DNA Gel Extraction Kit, Sangon, Shanghai, China). The purified fragments were then subjected to paired‐end sequencing with 27F and 1492R primers at Sangon Biotech Co. Ltd. (Shanghai). Sequence assembly was performed using BioEdit software, followed by manual correction. The corrected 16S rRNA sequences were compared with sequences in the NCBI database and the EzBioCloud database. Sequence alignment was conducted, and the strains were matched based on sequence similarity. Strains were matched to the species level if the similarity exceeded 99% with a single species, and to the genus level if the similarity exceeded 99% with multiple species.

### 2.5 Identification of VOCs Produced by the Metabolism of Antagonistic Bacteria

For the identification of VOCs, we selected single strains that showed better results in the non‐contact inhibition experiments. The methods chosen for identification include solid‐phase microextraction (SPME) and Gas chromatography‐mass spectrometry (GC–MS, Agilent 5975, USA). Antagonistic strains were cultured in 20 mL of LB liquid medium at 13°C with shaking at 200 rpm until reaching the logarithmic phase (OD600 of 0.8–1). A 20 μL culture suspension was spread on LB solid medium, with a blank control of LB solid medium without strain suspension. Both media were incubated at 13°C for 14 days, and the size of the petri dish is 90 mm in diameter and 18 mm in height. For VOC analysis, a SPME syringe with a 50/30 μm divinylbenzene/carbon in polydimethylsiloxane coating was conditioned at 270°C for 30 min. The medium was heated at 40°C for 30 min in a headspace vial to release VOCs with good thermal stability. The extraction head was then used to absorb VOCs for 20 min. GC–MS analysis was conducted with an initial temperature of 40°C (hold for 3 min), ramping to 180°C at 5°C/min, then to 230°C at 20°C/min (hold for 5 min). MS conditions included an interface temperature of 250°C, ion source temperature of 200°C, and EI ionization. VOCs were identified by comparing them to the National Institute of Standards and Technology database, excluding compounds found in the control.

A single VOC compound with high relative content was selected for validation of its inhibitory effect on *Pd*. SDA plates were inoculated with 100 μL of 6.5 × 10^5^/mL *Pd* spore suspension. Susceptibility tablets were placed on the lids of Petri dishes, which were then inoculated with 5 ppm of VOC standards. The plates were sealed with parafilm and incubated at 13°C for 14 days. Inhibition of *Pd* growth was observed and recorded.

## 3 Results

Seventy‐four bacterial strains were isolated, and a total of 18 isolates were tested for anti‐*Pd* effects through contact and non‐contact inhibition experiments (Table 1).

**TABLE 1:** The best match by sequence comparison with the database and the inhibitory effect of anti‐*P. destructans* strains isolated from *Rhinolophus ferrumequinum* wing membranes.

Label	16S rRNA					Inhibition effect		Degree of inhibition	
	NCBI		Ezbiocloud		The best match	Contact	Non‐contact	Reaction type	Mean score
	Similarity(%)	Bacterial taxa	Similarity (%)	Bacterial taxa					
3–1	99.88	*Pseudomonas carnis* (NZ_JANQAQ010000028)	98.43	*Pseudomonas carnis* (LT629744_s)	*Pseudomonas carnis*	√	√	E/E/E	5
3–8	99.58	*Pseudomonas carnis* (NZ_JANQAQ010000028)	99.50	*Pseudomonas carnis* (LT629744_s)	*Pseudomonas carnis*	√	√	D/D/D	4
3–9	98.18	*Pseudomonas carnis* (NZ_JANQAQ010000028)	98.22	*Pseudomonas paralactis* (KP756921)	*Pseudomonas* sp.	√		A/A/B	1.3
3–10	99.58	*Pseudomonas carnis* (NZ_JANQAQ010000028)	99.22	*Pseudomonas paralactis* (KP756921)	*Pseudomonas* sp.	√		C/C/C	3
2–3	98.36	*Pseudomonas carnis* (NZ_JANQAQ010000028)	99.22	*Pseudomonas paralactis* (KP756921)	*Pseudomonas* sp.	√		E/E/E	5
20–12	99.93	*Brevibacterium sanguinis* (NZ_MK176907)	99.84	*Brevibacterium aurantiacum* (X76566)	*Brevibacterium* sp.	√	√	A/B/A	1.3
19–6	99.64	*Brevibacterium aurantiacum* (NZ_CP025333)	99.72	*Brevibacterium aurantiacum* (X76566)	*Brevibacterium aurantiacum*	√		B/B/B	2
2–1	99.87	*Buttiauxella ferragutiae* (NZ_CP093332)	98.59	*Buttiauxella ferragutiae* (LXEQ01000068)	*Buttiauxella ferragutiae*	√	√	B/A/A	1.3
4–3	99.79	*Paraburkholderia fungorum* (NZ_BAYC01000000)	99.76	*Paraburkholderia fungorum* (BAYC01000104)	*Paraburkholderia fungorum*	√		B/D/E	3.7
19–1	99.80	*Arthrobacter rhombi* (NZ_FUHW01000049)	99.15	*Arthrobacter rhombi* (Y15885)	*Arthrobacter rhombi*	√		E/E/E	5
20–10	99.90	*Serratia liquefaciens* (NZ_CP048784)	99.76	*Serratia liquefaciens* (CP006252)	*Serratia liquefaciens*	√	√	E/E/E	5
19–8	98.31	*Brevibacterium aurantiacum* (NZ_CP025333)	98.40	*Brevibacterium zhoupengii* (MH915557)	*Brevibacterium* sp.	√		E/D/D	4.3
19–9	98.59	*Brevibacterium aurantiacum* (NZ_CP025333)	98.45	*Brevibacterium aurantiacum* (X76566)	*Brevibacterium* sp.	√		A/A/A	1
19–2	98.52	*Brevibacterium aurantiacum* (NZ_CP025333)	98.45	*Brevibacterium aurantiacum* (X76566)	*Brevibacterium* sp.	√		A/A/A	1
3–5	97.23	*Acinetobacter lactucae* (NZ_CP053391)	96.83	*Acinetobacter lactucae* (LRPE01000019)	*Acinetobacter* sp.	√		A/A/A	1
3–6	99.51	*Acinetobacter lactucae* (NZ_CP053391)	99.17	*Acinetobacter lactucae* (LRPE01000019)	*Acinetobacter lactucae*	√		A/A/A	1
3–13	99.58	*Acinetobacter lactucae* (NZ_CP053391)	99.10	*Acinetobacter lactucae* (LRPE01000019)	*Acinetobacter lactucae*	√		B/B/C	2.3
20–1	99.53	*Paeniglutamicibacter gangotriensis* (NZ_AOCK01000017)	99.62	*Paeniglutamicibacter gangotriensis* (AJ606061)	*Paeniglutamicibacter gangotriensis*		√	—	—

### 3.1 *Pd* Antagonistic Bacteria From Contact Inhibition Experiments

**FIGURE 1:**
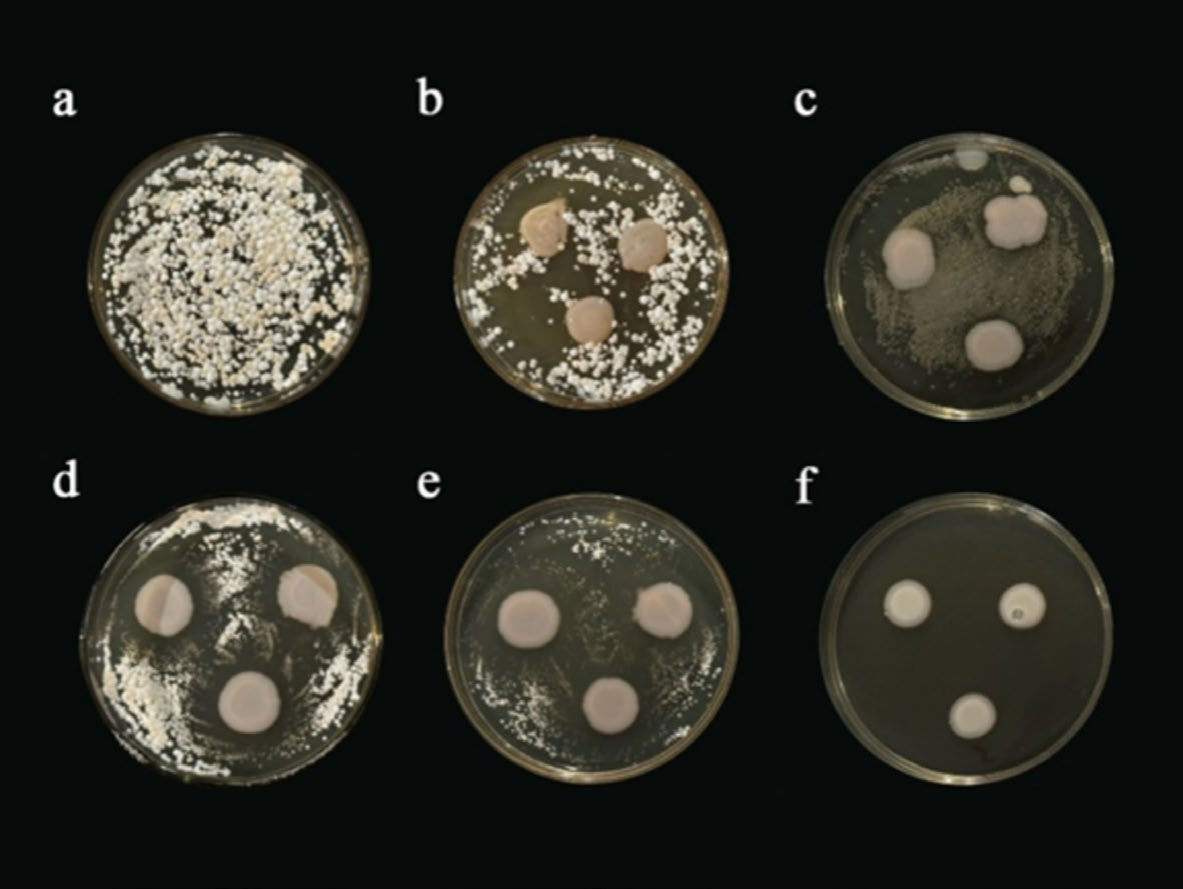
Anti‐*P. destructan*s activity in contact inhibition assays with skin‐isolated bacteria. (a) Control (only *P. destructans*); (b) Reaction type A: Isolated strains intermingle with *P. destructans* inhibiting one or both colonies; (c) Reaction type B: Inhibition halo < 2 mm, indicating limited inhibition within a short distance; (d) Reaction type C: Inhibition halo > 2 mm, indicating inhibition over a longer distance; (e) Reaction type D: Contact inhibition, where isolated strains grow unchanged or at a reduced rate through *P. destructans*; (f) Reaction type E: Distance inhibition, where the inhibitor grows through a clear zone with *P. destructans* at a reduced rate.

Seventeen isolates showed varying inhibition halos in the contact culture inhibition experiment (Figure 1). Nine isolates were identified as specific species based on 16S rRNA sequencing, including *Pseudomonas carnis*, *Buttiauxella ferragutiae* , *Paraburkholderia fungorum*, *Serratia liquefaciens* , *Arthrobacter rhombi* , *Brevibacterium aurantiacum* , and *Acinetobacter lactucae* (Table 1). The remaining 8 isolates were identified at the genus level, including *Pseudomonas*, *Brevibacterium*, and *Acinetobacter*. Different genera showed various colonial characteristics. For example, *Pseudomonas* colonies were round, approximately 2–3 mm in diameter, with a transparent, slightly pinkish center and a smooth, moist surface surrounded by an opaque, rough‐rimmed ring. *Brevibacterium* colonies were round, about 2 mm in diameter, creamy white, with a smooth, raised, transparent surface, and had a sticky feel when pricked with an inoculating needle.

**FIGURE 2:**
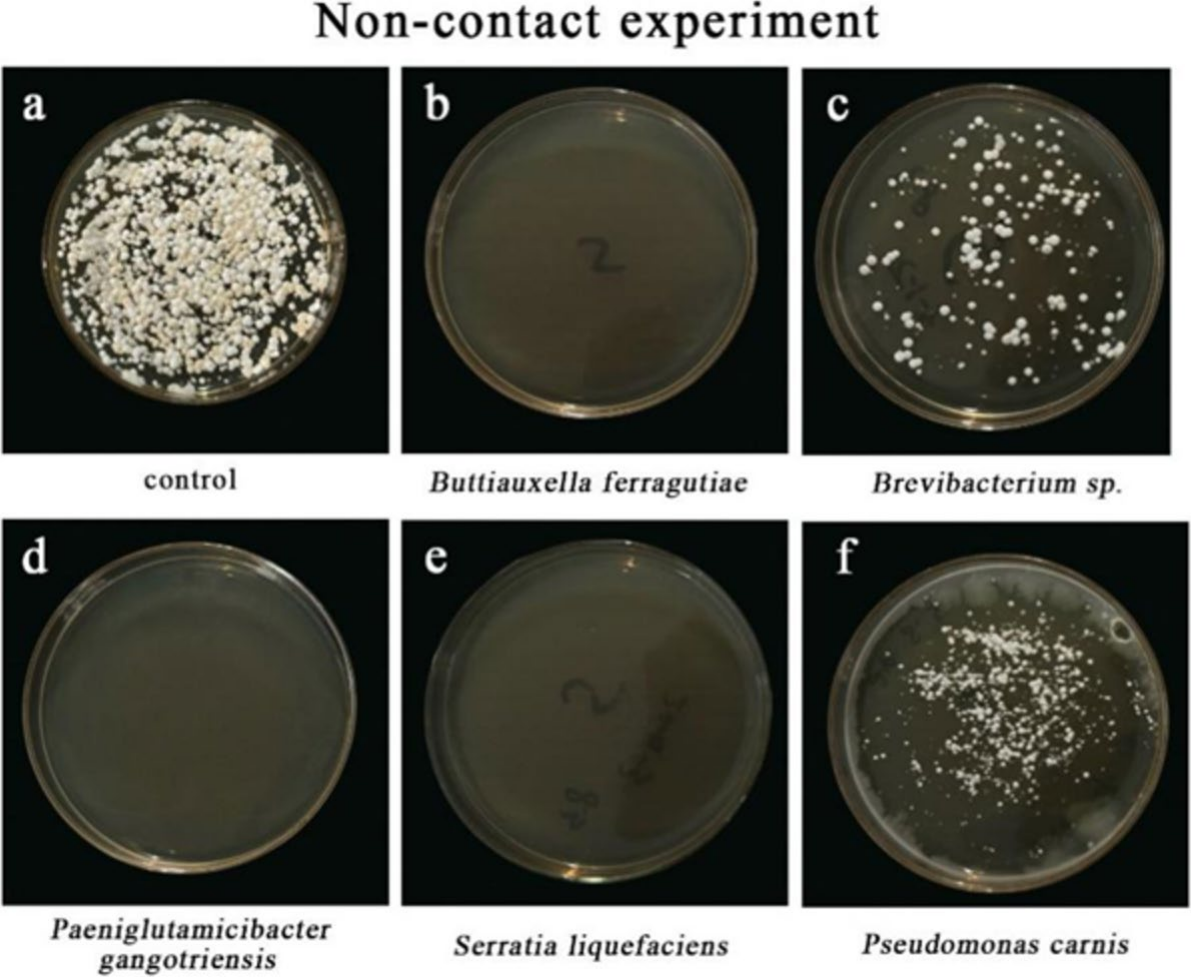
Evaluation of volatile organic compounds (VOCs) from skin‐isolated bacteria against *P. destructans*. (a) Control (only *P. destructans*); (b–f) Anti‐*P. destructans* activity in non‐contact inhibition assays with different skin‐isolated bacteria.

In contact inhibition assays, *P. carnis*, *S. liquefaciens* , *A. rhombi* , and an unidentified *Pseudomonas* strain (Table 1) effectively inhibited *Pd* at a distance (reaction type E, Figure 1f). Conversely, two *Acinetobacter* strains (*A. lactucae* and *Acinetobacter* sp.), and two unidentified *Brevibacterium* strains (Table 1) showed less inhibition (reaction type A, Figure 1b). Other strains exhibited moderate inhibition, ranging from reaction types D to B (Figure 1c–e). In non‐contact inhibition experiments, *B. ferragutiae* , *S. liquefaciens* , and *P. gangotriensis* achieved complete inhibition of *Pd* (Figure 2b), while two other strains partially inhibited *Pd* (Figure 2c).

### 3.2 *Pd* Antagonistic Bacteria From Non‐Contact Inhibition Experiments

In the non‐contact experiment, 6 isolates reduced *Pd* growth significantly compared to the control. Remarkably, 5 isolates exhibited both contact and non‐contact inhibition, indicating their effectiveness in different interaction modes (Table 1). Among these 5 isolates, four were identified to the species level: *P. carnis*, *B. ferragutiae* , and *S. liquefaciens* . One isolate was identified only to the genus level, *Brevibacterium*. In addition, the remaining isolate was identified as the specific species, *Paeniglutamicibacter gangotriensis*, which exhibited only non‐contact inhibition.

In non‐contact inhibition experiments, *B. ferragutiae* , *S. liquefaciens* , and *P. gangotriensis* achieved complete inhibition of *Pd* (Figure 2b,d,e), while two other strains partially inhibited *Pd* (Figure 2c,f).

### 3.3 Detection of Volatile Substances Produced by Antagonistic Bacteria

**FIGURE 3:**
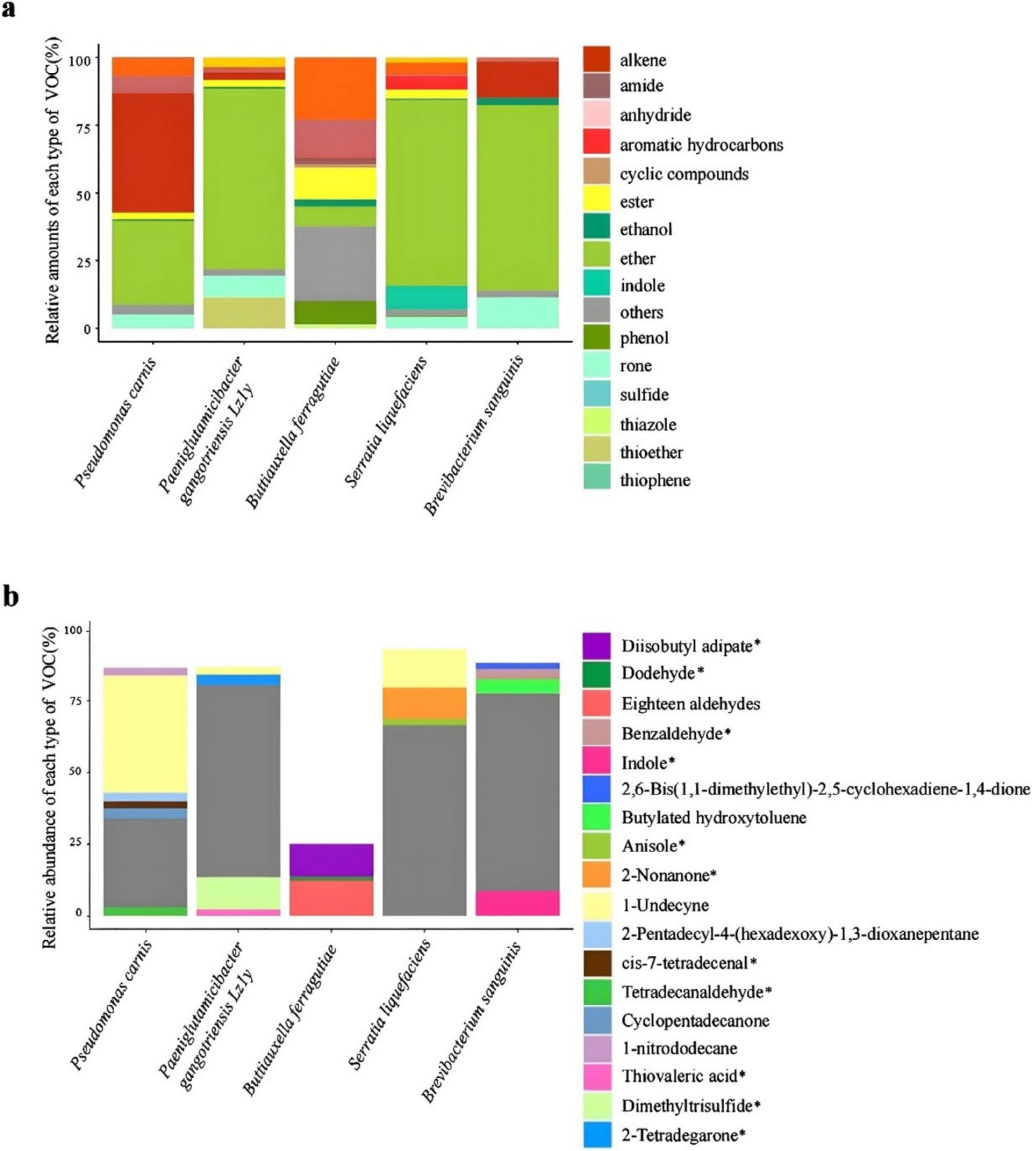
The types (a) and relative abundance (b, > 1%) of volatile compounds produced by anti‐*P. destructans* bacteria by no‐contact experiments. Compounds marked with an asterisk are compounds that are reported in the previous literature as having the ability to inhibit *Pd* (Table [Link], [Link]).

The VOCs produced by the five antagonistic strains with non‐contact inhibitory effects mainly included ketones, esters, alkenes, ethers, alkanes, alcohols, and aldehydes (Figure 3a). Ethers were the most abundant VOCs, making up between 31% and 69% of the total VOCs, except for *Brevibacterium* sp. Alkenes were notably high in *P. carnis* (44%) and *Brevibacterium* sp. (13%). Alkanes were found in significant amounts in *P. carnis* (6%) and *B. ferragutiae* (14%). The highest percentage of aldehydes was observed in *B. ferragutiae* (23%).

Based on the compounds with a relative abundance of more than 1%, differences in the types of VOCs produced by various antagonistic strain genera were observed (Figure 3b). Except for *Buttiauxella*, dimethyl disulfide is a significant VOC in the four genera, with relative abundances ranging from 31% to 69%. Indole, produced by *Brevibacterium*, is also a major VOC, with a relative abundance of 9%. Additionally, 2‐nonanone (11%) and 1‐undecene (13%) produced by *S. liquefaciens* are notable VOCs. Dimethyl trisulfide (11%) is the primary VOC produced by *P. gangotriensis*. Diisobutyl adipate (11%) and octadecanal (12%) are major VOCs produced by *Buttiauxella*. The content of 1‐undecene in *P. carnis* is as high as 41%. We found that 5 ppm of 1‐undecene, dimethyl trisulfide, and 2‐nonanone could completely or partially inhibit *Pd* growth (Figure S2b, c, d).

## 4 Discussion

Several bacterial genera, including both Gram‐positive (*Lactococcus*, *Bacillus*, *Paenibacillus*, *Rhodococcus*, and *Streptomyces*) and Gram‐negative (*Psychrobacter*, *Achromobacter*, *Serratia*, and *Pseudomonas*) strains, have been identified for their ability to inhibit *Pd* growth (Forsythe et al. 2022). In this study, nine antagonistic strains were isolated and matched to seven different species in contact inhibition. Five of these, excluding *P. fungorum* and *S. liquefaciens* , were isolated from bat wing membranes in western Canada and America, showing protection against *Pd* (Forsythe et al. 2022; Grisnik et al. 2020; Micalizzi et al. 2017). Notably, *P. fungorum* and *S. liquefaciens* were not previously reported for inhibiting *Pd*, but they have shown antagonistic effects on other fungal pathogens. For instance, *P. fungorum* inhibits certain plant pathogens and affects neighboring bacterial communities (Islam et al. 2023), while *S. liquefaciens* can lyse the mycelium of *Fusarium* spp., impacting its infectivity (Jia et al. 2023). In addition, eight unidentified isolates exhibiting contact inhibition were classified into the genera *Pseudomonas*, *Arthrobacter*, and *Brevibacterium*. *Pseudomonas* were found to inhibit fungal pathogens by altering plasma membrane properties and fluidity (Mahadevaswamy et al. 2024), with lipopolysaccharides triggering NF‐KBT signaling in fungal cells, leading to abnormal cell behavior (Li et al. 2024). *Arthrobacter* and *Brevibacterium* could also inhibit fungal pathogens through various mechanisms.

Several factors could contribute to contact inhibition effects. First, antagonistic strains may form biofilms in association with other microbial communities, which helps defend against fungal pathogens (Burmølle et al. 2006). Second, these strains might occupy adhesion sites on surfaces, thereby reducing fungal spore colonization (Kennedy and Volz 1985). Third, they could secrete biosynthetic products that alter their microenvironment, such as changing the pH (Matousek and Campbell 2002), creating conditions unfavorable for fungal growth. Additionally, antagonistic strains may produce active substrates or antifungal peptides to inhibit fungal pathogens (Bais et al. 2004; Boskey et al. 1999; Matousek and Campbell 2002; Ongena and Jacques 2008; Li et al. 2022; Sharma et al. 2025). For instance, *Pseudomonas yamanorum* produces phenazine‐1‐carboxylic acid, which inhibits *Pd* (Li et al. 2022). *Bacillus* sp. is known to secrete antifungal peptides, including β‐defensin‐like peptides, which can disrupt fungal cell membranes by electrostatic action (Lee et al. 2019; Sharma et al. 2025). Further studies using HPLC/MS could help identify active antifungal metabolites from these strains that are effective in contact inhibition of *Pd*.

In the non‐contact inhibition experiments, six antagonistic strains were isolated and identified, all of which released various VOCs with antifungal properties, and several of these compounds have been shown to have an inhibitory effect on fungal pathogens (Table S1). Diisobutyl adipate from *B. ferragutiae* significantly inhibited fungi like *Fusarium oxysporum* f. sp. *vasinfectum*, with increasing effectiveness at higher concentrations (Zhou et al. 2009). *Brevibacterium* strains produced butylated hydroxytoluene and indole, both showing strong inhibitory effects on fungi (Velikonja et al. 1987). Indole, a small molecule released by bacteria, regulates bacterial growth and biofilm formation, with properties effective against *Candida albicans* (Raut et al. 2012). *Serratia liquefaciens* released 2‐nonanone, which completely inhibits mango anthracnose pathogens and *Pd* at low concentrations (Zheng et al. 2013). *Pseudomonas gangotriensis* produced dimethyl trisulfide, known to inhibit multiple fungi, including *Aspergillus* sp. and *Penicillium* species, and also *Pd* (Kocic‐Tanackov et al. 2017; Lu et al. 2024). Additionally, dimethyl disulfide, produced by *P*. *carnis*, *B. sanguinis* , *S. liquefaciens* , and *P. gangotriensis*, was found effective against *Pd* (Hernández‐León et al. 2015). In this study, our experiments also demonstrated that 5 ppm of 1‐undecene, dimethyl trisulfide, and 2‐nonanone could completely inhibit *Pd* growth. These VOCs typically impede the growth and spore germination of pathogenic fungi, and affect mycelial morphology, mycotoxin production, enzyme activity, and gene expression (Herrera‐Cabrera et al. 2024; Ongena and Jacques 2008; Tyagi et al. 2020).

This study underscores the significant role of skin microbiota in inhibiting *Pd* growth through both contact and non‐contact inhibition modes. However, beyond skin microbiota, host‐specific factors, such as frequent grooming behavior (Neubaum and Siemers 2021) or the production of antifungal peptides, also play crucial roles in defending against fungal pathogens (Cheng et al. 2021; Langwig et al. 2015). Therefore, future research should integrate multiple disciplines to explore the comprehensive inhibitory mechanisms underlying bats' resistance to *Pd*.

## 5 Conclusion

In this study, 18 antagonistic bacteria inhibiting *Pd* growth were isolated from *R. ferrumequinum* skin. Of these, 17 exhibited contact inhibition, while 7 showed non‐contact inhibition, and they were identified as belonging to 12 taxa. Among those exhibiting contact inhibition, *P. carnis*, *A. rhombi* , *S. liquefaciens,* and an unidentified *Pseudomonas* species were most effective. For non‐contact inhibition, *B. ferragutiae* , *S. liquefaciens* , and *P. gangotriensis* were most effective. Approximately 20 VOCs released from those antagonistic strains exhibiting non‐contact inhibition were detected, most of which have antifungal properties. For example, 5 ppm of 1‐undecene, dimethyl trisulfide, and 2‐nonanone showed significantly inhibitory effects on *Pd*. This research highlights the skin microbiota as a crucial defense against fungal pathogens and may offer new approaches and microbial sources for controlling *Pd* spread.

## Author Contributions

**Yanqing Da:** software (equal), validation (equal), visualization (equal), writing – original draft (equal). **Mingxuan Liu:** validation (equal), writing – original draft (equal). **Yangshuang Zhu:** validation (equal). **Weixu Wang:** software (equal), validation (equal). **Yaping Lu:** investigation (equal), validation (supporting). **Keping Sun:** funding acquisition (lead), methodology (lead), writing – review and editing (supporting).

## Ethics Statement

The field work was approved by the Science and Technology Ethics Committee of Northeast Normal University, China (approval number NENU‐2019‐04).

## Conflicts of Interest

The authors declare no conflicts of interest.

## Supporting information


**Figure S1.** (a) map of the sampling site; (b) picture of the cave’s external environment; (c) picture of hibernating bats inside.

**Figure S2.** Inhibition effects of three compounds on *Pd* at 5 ppm concentration. (a) control (*P. destructans* only); (b) 2‐nonanone at 5 ppm; (c) 1‐undecene at 5 ppm; (d) dimethyl trisulphide at 5 ppm.

**Table S1.** Comparison of previously reported VOCs capable of inhibiting *Pd* with the compounds identified in this study.

## Data Availability

All 16S rRNA sequence data (SUB14527932) have been deposited in the National Center for Biotechnology Information sequence read archive (https://www.ncbi.nlm.nih.gov/).
