# Supplementary figures and images for: Isolation of Antagonistic Bacterial Strains and Their Antimicrobial Volatile Organic Compounds Against Pseudogymnoascus destructans in Rhinolophus ferrumequinum Wing Membranes
Source: Ecol Evol. 2025 Jun 27;15(7):e71628. doi: 10.1002/ece3.71628 (PMC12204724; doi:10.1002/ece3.71628)

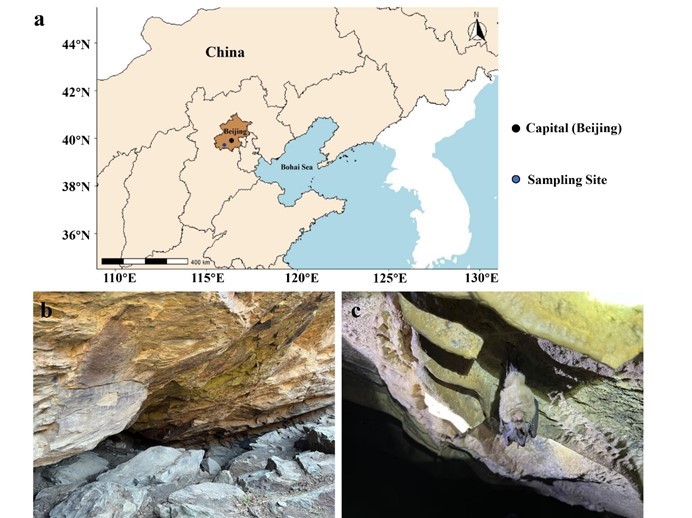

Supplement: Supplementary file 1 — Figure S1. (a) map of the sampling site; (b) picture of the cave’s external environment; (c) picture of hibernating bats inside. [file ECE3-15-e71628-s001.jpg]

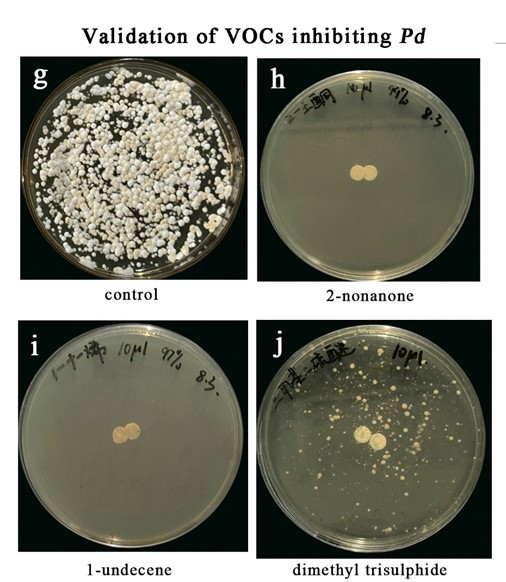

Supplement: Supplementary file 2 — Figure S2. Inhibition effects of three compounds on Pd at 5 ppm concentration. (a) control (P. destructans only); (b) 2‐nonanone at 5 ppm; (c) 1‐undecene at 5 ppm; (d) dimethyl trisulphide at 5 ppm. [file ECE3-15-e71628-s002.jpg]
