# Supplementary material for: Isolation of Antagonistic Bacterial Strains and Their Antimicrobial Volatile Organic Compounds Against Pseudogymnoascus destructans in Rhinolophus ferrumequinum Wing Membranes
Source: Ecol Evol. 2025 Jun 27;15(7):e71628. doi: 10.1002/ece3.71628 (PMC12204724; doi:10.1002/ece3.71628)
Supplement: Supplementary file 3 — Table S1. Comparison of previously reported VOCs capable of inhibiting Pd with the compounds identified in this study. [file ECE3-15-e71628-s003.docx]

**Table S1.** Comparison of previously reported VOCs capable of inhibiting *Pd* with the compounds identified in this study

| **Classification of VOCs** | **Specific classification** | **Reported previously** | **Found in this study** |
| --- | --- | --- | --- |
| Aldehydes | Alkyl aldehyde | Acetaldehyde (Gabriel et al., 2018) | Eighteen aldehydes |
|  |  | Aonanal (Cornelison et al., 2014) | Tetradecanaldehyde |
|  |  | Decanal (Cornelison et al., 2014) |  |
|  | Alkenyl aldehyde | 2-Hexenal (Padhi et al., 2018) | Cis-7-tetradecena |
|  |  | 2-Butenal, 2-methyl-, (E)- (Gabriel et al., 2018) |  |
|  | Aromatic aldehyde | Benzaldehyde (Cornelison et al., 2014) | Benzaldehyde |
| Ketone | Alkyl ketone | 2-Butanone (Gabriel et al., 2018) | 2-Nonanone |
|  |  |  | 2-Tetradegarone |
| Esters | Alkyl aldehyde | Methyl ester (Gabriel et al., 2018) |  |
|  |  | 2-Methylbutyl ester(Gabriel et al., 2018) |  |
|  | Acid aldehyde | Acetic acid, 2-methylpropyl ester (Gabriel et al., 2018) | Diisobutyl adipate |
|  |  | 2-methyl-, 2-methylpropyl ester (Gabriel et al., 2018) |  |
|  |  | 1-Butanol, 3-methyl-, acetate (Gabriel et al., 2018) |  |
| Sulfhydryl substitutes | Sulfur ether | Dimethyl disulphide (Li et al., 2022; Lu et al., 2024) | Dimethyl disulphide |
|  |  |  | Dimethyl trisulphide |
|  | Thioctic acid |  | Thiovaleric acid |
| Alcohol / Ether | Alcohol | 1-Propanol, 2-methyl- (Gabriel et al., 2018; Micalizzi et al., 2017; Micalizzi & Smith, 2020) |  |
|  |  | 1-Butanol, 3-methyl- (Gabriel et al., 2018) |  |
|  |  | 2-ethyl-1-hexanol (Cornelison et al., 2014) |  |
|  |  | 2-methyl-1-butanol (Micalizzi et al., 2017; Micalizzi & Smith, 2020) |  |
|  |  | 1-pentanol (Micalizzi et al., 2017; Micalizzi & Smith, 2020) |  |
|  |  | 1-octen-3-ol (Padhi et al., 2018) |  |
|  | Ether |  | Anisole |

**References**

Cornelison, C. T., K. T. Gabriel, C. Barlament, and S. A. Crow Jr. 2014. “Inhibition of *Pseudogymnoascus Destructans* Growth From Conidia and Mycelial Extension by Bacterially Produced Volatile Organic Compounds.” *Mycopathologia* 177, no. 1–2: 1–10. <https://doi.org/10.1007/s11046-013-9716-2>

Gabriel, K. T., D. Joseph Sexton, and C. T. Cornelison. 2018. “Biomimicry of Volatile‐Based Microbial Control for Managing Emerging Fungal Pathogens.” *Journal of Applied Microbiology* 124, no. 5: 1024–1031. <https://doi.org/10.1111/jam.13667>

Li, T., L. Li, F. Du, et al. 2021. “Activity and Mechanism of Action of Antifungal Peptides From Microorganisms: A Review.” *Molecules* 26, no. 11: 3438. <https://doi.org/10.3390/molecules26113438>

Li, Z., A. Li, J. R. Hoyt, et al. 2022. “Activity of Bacteria Isolated From Bats Against *Pseudogymnoascus Destructans* in China.” *Microbial Biotechnology* 15, no. 2: 469–481. <https://doi.org/10.1111/1751-7915.13765>

Lu, Y., H. Ren, Z. Li, et al. 2024. “Microbiota Diversity and Anti‐*Pseudogymnoascus Destructans* Bacteria Isolated From *Myotis Pilosus* Skin During Late Hibernation.” *Applied and Environmental Microbiology* 90, no. 8: e0069324. <https://doi.org/10.1128/aem.00693-24>.

Micalizzi, E. W., and M. L. Smith. 2020. “Volatile Organic Compounds Kill the White‐Nose Syndrome Fungus, *Pseudogymnoascus Destructans*, in Hibernaculum Sediment.” *Canadian Journal of Microbiology* 66, no. 10: 593–599. <https://doi.org/10.1139/cjm-2020-0071>

Micalizzi, E. W., J. N. Mack, G. P. White, T. J. Avis, and M. L. Smith. 2017. “Microbial Inhibitors of the Fungus *Pseudogymnoascus Destructans*, the Causal Agent of White‐Nose Syndrome in Bats.” *PLoS One* 12, no. 6: e0179770. <https://doi.org/10.1371/journal.pone.0179770>.

Padhi, S., I. Dias, V. L. Korn, and J. W. Bennett. 2018. “*Pseudogymnoascus Destructans*: Causative Agent of White‐Nose Syndrome in Bats Is Inhibited by Safe Volatile Organic Compounds.” *Journal of Fungi* 4, no. 2: 48. <https://doi.org/10.3390/jof4020048>.
